# Belt and Road Environmental Implications for South Asia

**DOI:** 10.3389/fpubh.2022.876606

**Published:** 2022-04-25

**Authors:** Majid Ali, Khan Faqir, Bilal Haider, Khurram Shahzad, Nosheen Nosheen

**Affiliations:** ^1^School of Marxism, Xi'an Jiaotong University, Xi'an, China; ^2^Pakistan Study Centre, University of Peshawar, Peshawar, Pakistan; ^3^School of International Law, Chinese University of Political Science and Law, Beijing, China; ^4^Department of History, University of Peshawar, Peshawar, Pakistan

**Keywords:** Green Belt Road, CPEC, green technology, Paris agreement, South Asia

## Abstract

The Belt and Road Initiative (BRI) can play a significant role in the sustainable development of South Asia if appropriately implemented. Apart from the economic, trade, and cultural benefits of this colossal infrastructure, less is known about its environmental impact on South Asia. This study looks closely at the potential impact of the BRI on the South Asian environment. This research is based on the government-issued environmental policies, peer-reviewed literature, media articles, and reports. It has been suggested that the BRI could have a negative impact on the South Asian environment, which provided if does not consider the Paris agreement with its partners in the region. The study suggests that partner countries should adopt the BRI project to the principles of environmental impact assessment.

## Introduction

Belt and Road Initiative (BRI) is an unprecedented investment of multi-billion dollars over 4.4 billion people of 138 countries connecting China with Europe through road and sea, such as the ancient Silk Road. The literature has discussed its economic, geopolitical, and cultural implications in detail in the context of South Asia, but less attention has been paid to its environmental implications. Its activities (methods, practices, technologies, and chemicals) can be hazardous or beneficial to the environment of South Asia.

It is fact that the first step of the whole BRI project is construction, whether these are roads, rail lines, airports, seaports, dried ports, special economic zones (SEZ), industrial parks, excavations, minings, gas pipelines, cement productions, or steel productions. All infrastructural and constructional activities directly or indirectly affect the environment except Digital Silk Road, Clean Silk Road, and Green Silk Road. Besides that, mega constructions are full of risks and uncertainties qualitatively and quantitatively and produce a considerable volume of waste and energy emissions, which are harmful to the environment (1).

World Bank report says about BRI environmental impact that “such kind of large transportation projects exposes countries and local communities to environmental and social risks as BRI routes passing through Cambodia-Kyrgyzstan-Laos increased carbon dioxide (CO_2_) emission 7% in total [(2), p. 7].” Considering that BRIs are still spreading status, the researchers, Governmental and non-Governmental organizations are worried about its environmental implications. For South Asian partner countries, it will be a challenge to foster their economies while protecting their environment by applying policies such as “Green Belt and Road” and “Ecological Civilizations” as adopted by China (3).

It is about ever-growing scholarship on BRI's environmental aspect that did not heed opportunities associated with environmental challenges. This paper aims to contribute to the extant literature and debates about the ecological impact of BRI on South Asia. The specific focus is to investigate the environmental gray areas and suggest potential green technology (GT) and policies to the partner countries to counter the negative impact of BRI.

The article is divided into four sections to know the implications of BRI in South Asia. The first section reviews the overall environment of South Asia. Second, it assesses China's domestic environmental initiatives and its impact. Third, it highlights BRI's impact on the environment of South Asia. Fourth, it elaborates the finding's drawbacks and provides policy-related recommendations to partner with China to bridge environmental components with construction.

## Research Questions

What are the possible Positive and negative impact of construction? Then, how South Asian Economies can avoid negative impacts of developmental works while carrying on BRI projects?

## BRI and South Asia Need and Importance

In spite of the high potential capabilities of business and trade of South Asian countries, the unavailability of cross-border infrastructure, non-connectivity, ineffective trade, and the lack of cross transit transport facilities led the countries to their minimum advantage [(4), p. 184]. Back in 2009, Asian Development Bank (ADB) suggested in a study that from 2010 to 2020, Asia needs to invest more than $8 trillion in transportation, power, telecommunication, water supply, and sanitation on average $750 billion per year in more than 32 countries out of 45 countries of Asia [(5), p. vii]. Then in 2017, ADB suggested $26 trillion for Asian development until 2030. In which $14.7 trillion were mentioned for infrastructure, $8 trillion for transportation, $2.3 trillion for telecommunication, $800 billion for water and sanitation, and remaining for energy and power [(6), p. 6], [(5), p. vii]. World Bank made it clear that if the infrastructure is available and the investors are interested, then prosperity is inevitable, “the largest source of external finance in many developing countries is an investment which has potential to drive significant growth, it facilitates technological transfer, promote market competition can create stable jobs, enhance productivity and can provide a conducive environment for home investments [(7), p. 17)].” One of the studies mentioned that infrastructure is the backbone of mobility, every country requires it, even if it is developed or underdeveloped, like if Germany today did not patch to the idea of enhancing its infrastructure, then it may lose its standard in the race of industrialization, and if it joins BRI, it could renew its infrastructure as China did [(8), p. 12].

The infrastructure and foreign investment helped the developed countries, and they took advantage of free trade and investments in infrastructure development and its maintenance [(9), p. 301]. Asia can do the same with Chinese and other investments, because it is becoming an essential destination for the Global economy due to its fast growth potential. Asia needs peaceful development for its economic revitalization, and for that, BRI is the right choice at the right time [(10), p. 63]. Then, according to World Bank, the BRI can be beneficial for job creation and local skill development, especially in South Asia [(7), p. 6]. The assumption of WB was verified with numbers when President Xi Jinping confirmed that “BRI created more than 200000 local jobs in the partner countries [(11), p. 61].”

It is assumed that BRI transport projects will increase trade between 2.8 and 9.7% with the partner countries and 1.7 and 6.2% for the whole world [(2), p. 14]. China is currently contributing 30% of share to the total global infrastructure, and according to the Global Infrastructure Hub (GI-Hub), it is a moderate investment, with continuity and fluctuations between electricity and rail infrastructure. Chinese investment is similar to the Global required average investment [(12), p. 76]. Although demand for global infrastructure is increasing day by day, and by 2040 globally, there will be a need for more than $94 trillion for infrastructural development [(13), p. 180]. World Bank further mentioned that “BRI transport infrastructure can reduce travel times for economies along the affected transport corridors by up to 12 percent, reducing trade costs and increasing trade by an estimated 2.8% to 9.7% for corridor economies and 1.7–6.2% for the world [(7), p. 5].” Keeping in view the need and expectation of BRI, one can say that it provides opportunities for trade and investment. It will also create jobs and increase consumption; besides that, it will develop infrastructure and political association among China and partner countries. It will have a significant impact on the economic growth of Asian Economies along with other macroeconomic variables, such as imports, political stability, and corruption (14). No doubt it may bring dramatic changes to the life of unprivileged people, create opportunities for better health, develop educational services, and may improve social conditions, but such types of activities may also bring their challenges, such as sustainability, climate change, pollution, and other environmental implications [(5), p. 3]. That is why detailed work is necessary to carry out its environmental impact.

Before going into detail about the environmental implications of BRI for South Asia, our paper tried to explain the difference between Clean Silk Road and Green Silk Road. The earlier is an initiative to control malpractices and ensure transparency. In 2019, a Thematic Forum on Clean Silk Road of the second Belt and Road Forum for International Cooperation was held in Beijing, with the motto to fight against corruption under United Nations Convention for BRI projects [See for detail (69)]. Green Silk Road, as its name, is conveying greenery. It tries to make all the activities under BRI environment friendly. Xi Jinping Outlined Green Silk Road in these words “We should uphold the concept of green development, advocate a low-carbon, recyclable and sustainable lifestyle, and strengthen ecological cooperation to make it a part of our life so that we can join hands in achieving the United Nations Sustainable Development Goals by 2030 [See for detail (15)].”

### Overall Environment Issues of South Asia

**Figure 1:**
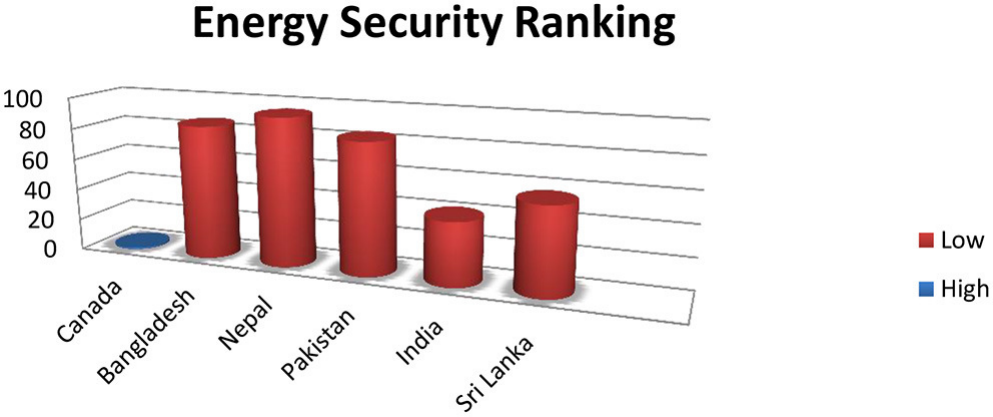
Higher numbers denoting insecurity, compared with Canada: (16).

Here, we present the region's environmental sustainability, energy security, and energy equity, to give us a clear picture of South Asia's BRI needs and anticipated risks. The energy security (the resilience of energy infrastructure) of South Asian countries is shallow; out of 101 countries, Bangladesh is 85, Nepal 94, Pakistan is 83, India is 40, Sri Lanka is 55, and at the topper of the ranking is Canada, which is number 1 (Figure 1).

**Figure 2:**
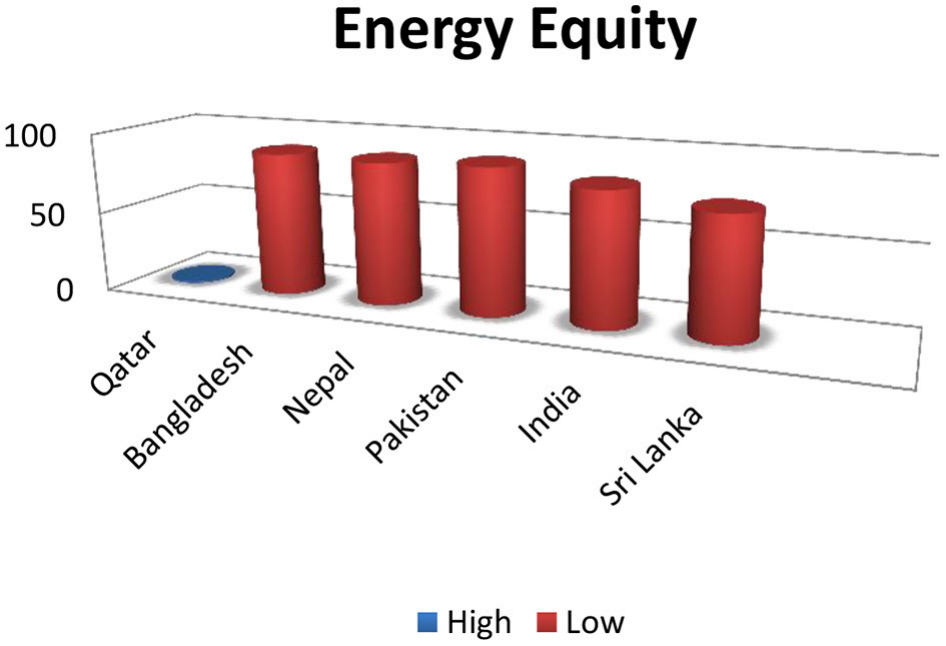
Higher numbers denoting insecurity, compared with Qatar: (16).

South Asian countries, according to the World Economic Council (WEC), criteria for energy equity (which is the provision of universal access to reliable and affordable energy for home and commercial use), are lower in rankings, Bangladesh 89, Nepal 88, Pakistan 90, India 82, and Sri Lanka 74, respectively. The topper in the list is Qatar, with number 1 (Figure 2). This chart shows that the mentioned countries are in dire need of energy for their domestic use.

**Figure 3:**
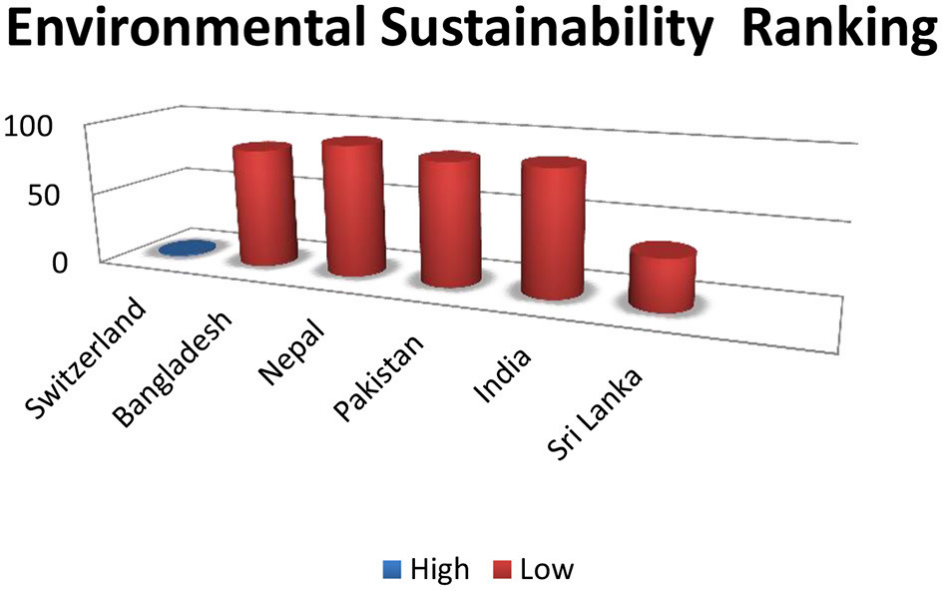
Higher numbers denoting insecurity, compared with Switzerland: (16).

South Asia for environmental sustainability is at high risk because it emits numerous gases that affect air and water. The energy consumption of South Asian countries is not environmentally friendly and directly impacts climate change (the detailed discussion in BRI and South Asia's need and importance). Here, we showed a comparative environmental ranking of South Asian Countries (Figures 3, **5**).

**Figure 4:**
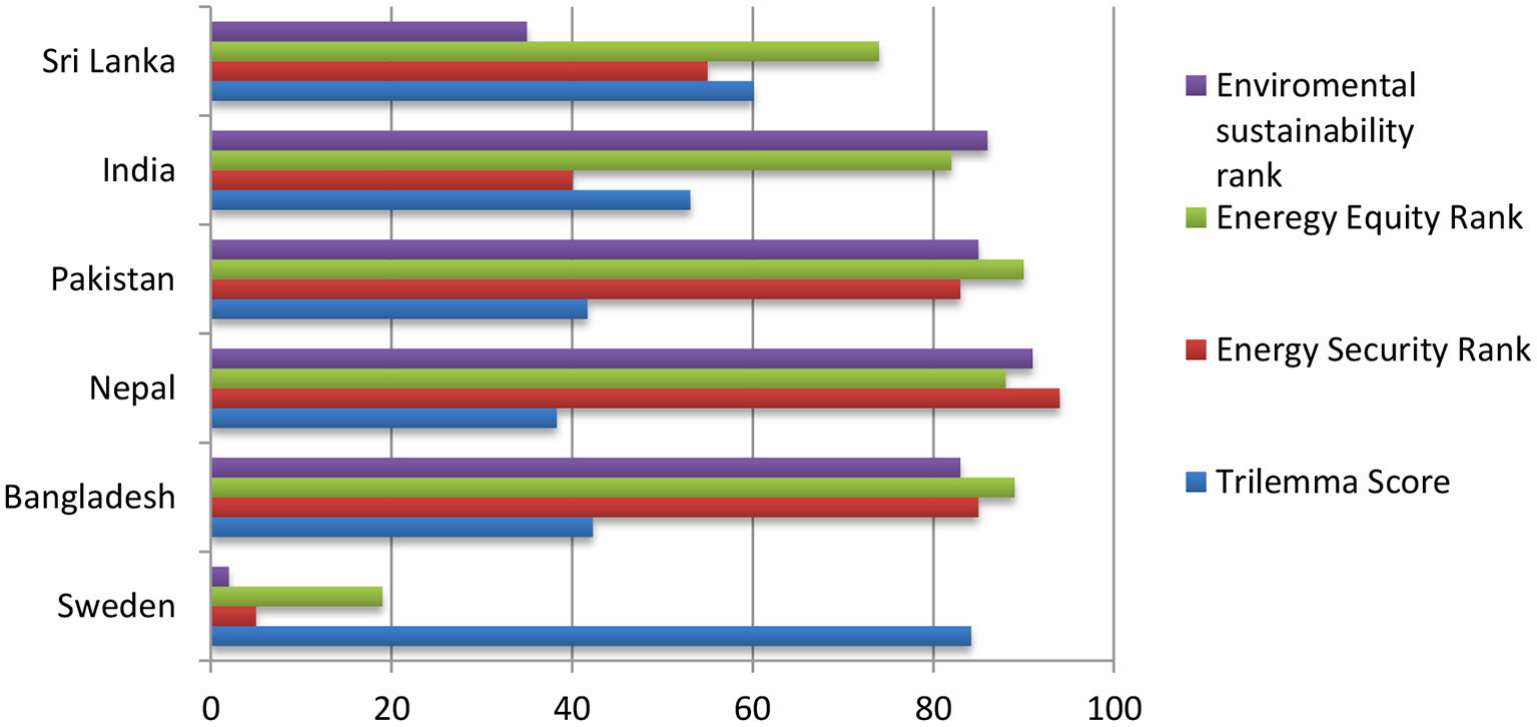
Countries comparative chart of trilemma score.

South Asian Countries in the World Energy Council list ranking of 2021 are at 87 Bangladesh, 96 Nepal, 90 Pakistan, 75 India, and 60 Sri Lanka. The first country for the overall ranking is Sweden, with an 84.2 score in the 5th trilemma (healthy energy the system, which is equitable and environmentally sustainable and carefully managed) on energy security rank 19th on energy equity ranking, and 2nd on environmental sustainability rank [(16), Figure 4].

**Figure 5:**
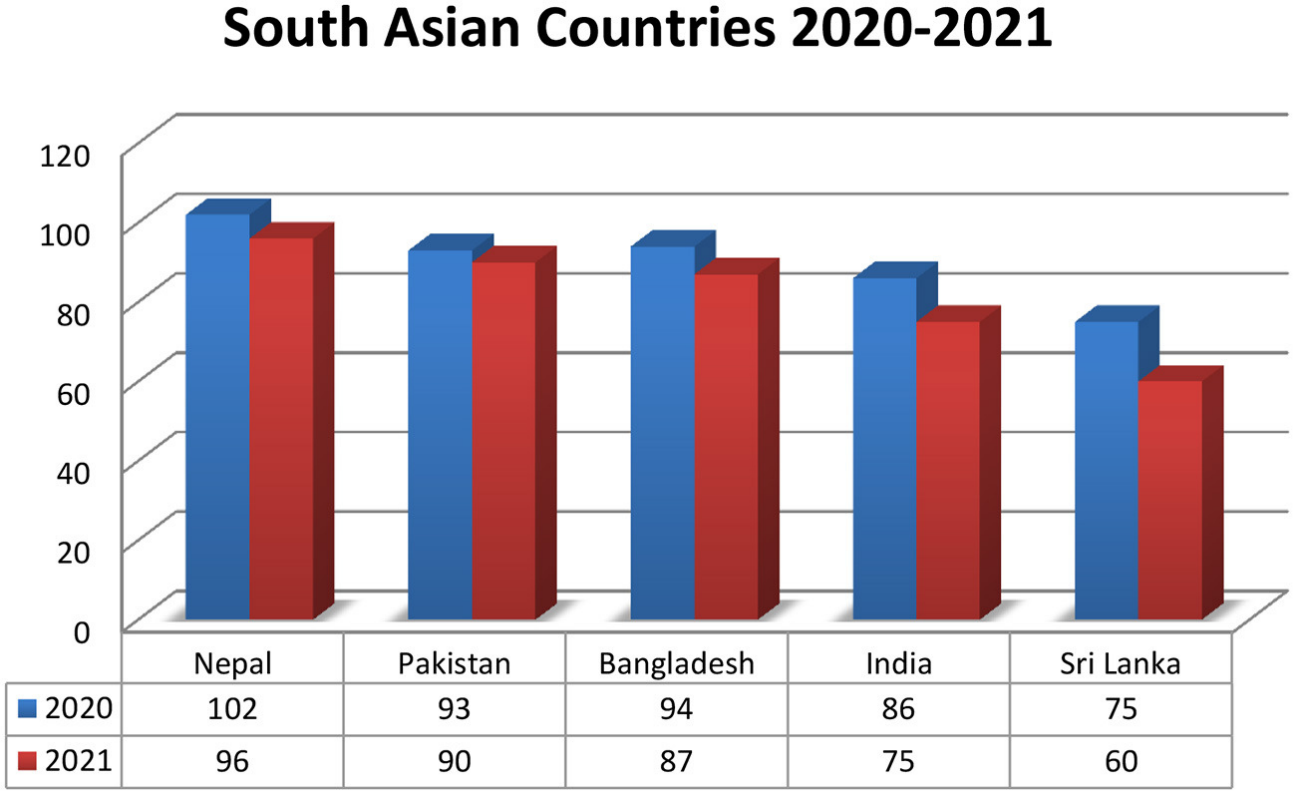
Countries year-wise comparision (2020–2021).

As compared to 2020, the year 2021 showed progress of South Asian countries (Figure 5).

**Figure 6:**
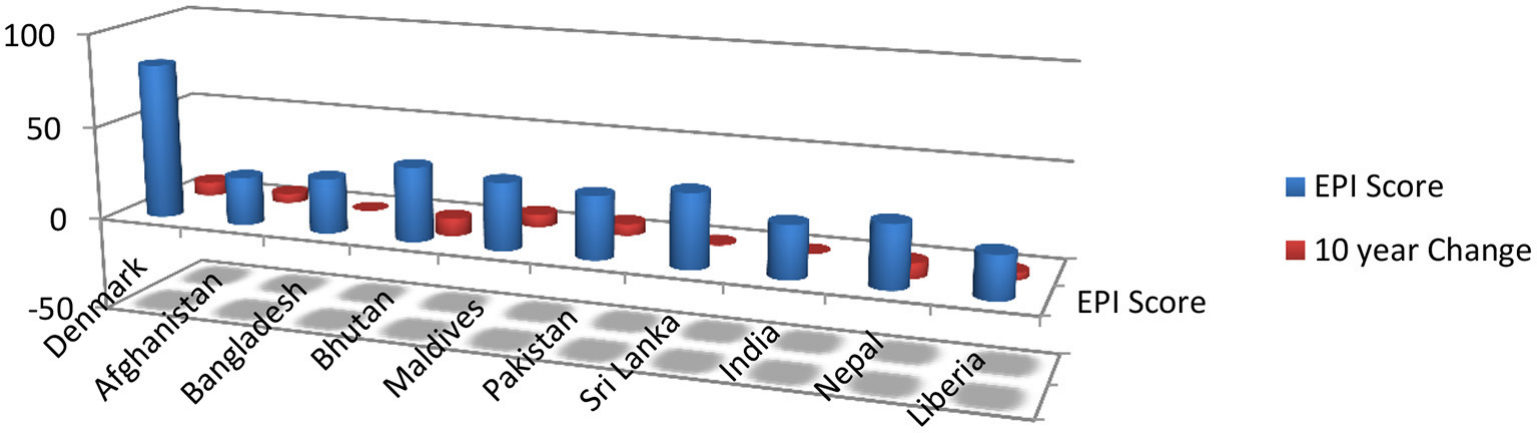
South Asian Environment Performance Index (18).

It is the fact that the environment has been affected by economic development. First, it deteriorates the environment by economic growth, and then, it improves the environmental effects by improving technologies, rules, techniques, and forestations. It is expected same from south Asia (17). Before going into detail, we put here the present situation of the South Asian environment through a comparative analysis chart. According to the comparative Environmental Performance Index (EPI), South Asian countries are prone to risks. Out of 180 countries, South Asian countries rank between 107 and 178 [Figure 6: (18)].

Here, we share the point of view of one of the respondents of a qualitative study, who concluded that out of total BRI projects, 22% is Hydropower, 19% is rail infrastructure, and 19% is manufacturing, which is highly prone to the negative impact on the environment (19).

### China Domestic Environmental Initiatives

Chinese policy about the green environment is evident, which is mentioned by Xi Jinping to the participants of the Belt and Road Forum for International Cooperation in Beijing “We should peruse the new vision of green development and a way of life and work that is green, low-carbon, circular and sustainable.” Efforts should be made to strengthen cooperation in ecological and environmental protection and build a sound ecosystem to realize the goals set by the 2030 Agenda for Sustainable Development (SDG) [(20), p. 252]. GT, innovative ideas, green trade, financing, and investment in green growth are some mechanisms that can accelerate achieving SDGs. Sources claim that the Chinese government is determined to provide financial help in implementing the Paris agreement to BRI countries (3).

But the question is how many countries of South Asia are intends to implement Paris agreement for green agendas. Xi Jinping made a speech to the Central Committee of the Communist Party of China for the 13th five-year plan, for economic and social development, at the Fifth Plenary session of the 18th CPC Central Committee in October 2015, where he said that we must promote effective control of ecology and address growing resources constraints, serious environmental pollutions, and ecological degradation. He further explained that “we need to save energy, water, land resources, reduce pollutant emissions and raise the growth level model by Green Economy [(21), p. 421].” It gives a complete picture to the BRI partner countries of South Asia that they can also consider the “dual control” and avoid hazardous emissions. The green shift is necessary to develop a beautiful China by phasing out of polluting industry and reducing emissions by restoring degraded ecosystems (22). By passing the environmental protection act and imposing a ban on China's natural forests in northeastern provinces (22).

## The Impact of the Chinese In-Land Environmental Policies

Chinese investment in environmental regulations can induce innovation in cleaner technologies (23). Some Chinese banks under the environment protection bureau can halt the loans or grants if their activities threaten unfavorable environmental outcomes for other countries. That is how they can reduce water pollution. One study found that firms were compelled to use end-of-pipe treatment since the imposition of this policy, long-term pollution, can be constrained (24). China uses too much fertilizer to produce enough food to feed 20% of the world's population. However, that resulted in air and water pollution. Some policies were adopted to counter exacerbated use of fertilizers and their non-organic nutrients. Fortunately, that showed a positive change in some municipalities such as Shanghai 2004, Jiangsu 2006, Beijing 2007, and Shandong 2008. They enhanced organic waste utilization, which reduced pollution. The government started more than 15 such initiatives for green manures and organic fertilizers (25).

### BRI Impact on the Environment of South Asia

Belt and Road Initiatives will increase the environmental crisis in already vulnerable environmental geographies (Figure 3) by increasing the air and water pollution and may create water shortage and soil erosion so severely that it can reduce the life span of the participant country's population (22). Nevertheless, some studies argue that BRI will use excessive raw materials for its large-scale infrastructure project, which may directly impact the environment (19).

**Figure 7:**
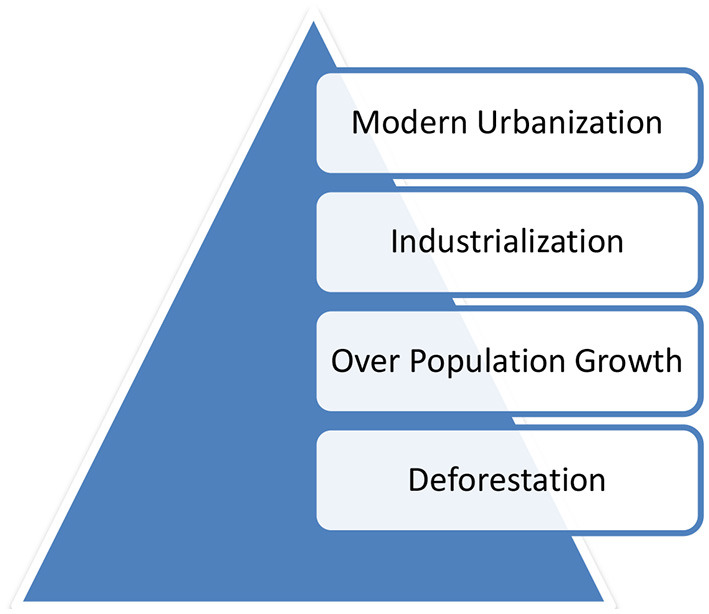
Triangle of environment causes.

Another study concluded that continues economic growth is not favorable to green growth (26). Raising how economic growth can occur in under-developed or South Asian countries of BRI can be favorable to green growth. The recent work categorizes the primary reasons for environmental degradation in chart form (27) (Figure 7).

#### Infrastructural Project Impact

The physical infrastructure directly impacts soil, air, medicinal plants, and habitats; deforestation, emission of transportation, and use of chemicals for construction purposes are decreasing the greenhouse effect (3). Construction without BRI is also going on in all countries. It is neither exhaustive nor exclusive, but construction without environmental care may impact related and non-related countries, demanding environmental friendly policies.

However, the damage to the environment has been caused by air pollution, climate change, shortage of natural resources, and increasing waste generation, particularly from the construction industry. These constructions contribute ~36% of total waste globally (28). South Asian countries have a poor history of road construction, while in addition, the construction at hillsides saw rock-falling, landslides, debris flow, snow avalanches, and road closers. That all happened during the construction of the Panj River in Afghanistan; however, it is not found that these caused any deaths or property damages, but it is found that landslides, debris flows, and rock-fall may damage the mountainous roads (29). In the light of construction, it is found by a policy study that 40% of energy is being consumed by the real estate sector of total global energy, that is why it is producing 20% of greenhouse gas emissions around the globe, and by 2030, such kind of emission will reach up to 56% (30).

#### Traffic, Gas Emission, and Its Environmental Impact

Due to traffic, there may be massive pollution of air and noise depending upon the country's transportation usage. However, electric-powered rails may lower air pollution and greenhouse gas emissions if BRI particular roads are used. On the other hand, the existing roads in BRI countries did not have the capacity for green transportation [(2), p. 111]. How the roads and its traffic under BRI will affect the environment in hilly areas has been portrayed by one of the scholars in these words that “A truck carries Goods from China through poorly maintained Pamir Highway (M51) to Tajikistan, will block the road while creating noise and dust pollution, is an addition to the already environmentally challenged area (29).”

China is moving toward a green economy by imposing bans on environmentally risky activities such as deforestation and polluting industries. However, it produces chances for China to outsource these industries or relocate to the partner countries, which is not under Chinese environmental laws jurisdiction and are contagious for ecological pollution (22). It needs more empirical research to prove that the flow of polluted air is not going into the pollution-free area; otherwise, Chinese efforts of a green economy will benefit less than they are expecting. It may cause depletion of resources of all biotic and abiotic elements, which includes air, water, soil, plants, animals, and other living and non-living elements of planet earth (27).

The poisonous gases such as chlorofluorocarbon (CCl_2_ F_2_), nitrogen oxide (NO), and carbon monoxide (CO) sent to the environment are sent by industries and automobiles, which will be increased in the coming days due to the BRI projects. However, it will add more pollution to the already polluted air of South Asia because BRI will not only increase the economic activities but also relocate people to developed urban facilities. One of the studies found that urban population of 10% causes 24.7% emission of household carbon as compared to the whole 46% population in China, which causes emission 24.6% and that will increase till 2050 with the distribution of carbon inequality (31). Another study supported this logic in these words that “due to the rapid Urbanization and Industrialization, air pollution may emerge as a significant global concern in decades, which may have adverse impacts on health (32).”

For air quality, out of 106 countries, South Asian countries are on top, Bangladesh 1, Pakistan 2, India 3, Afghanistan 5, Nepal 12, and Sri Lanka is on 30 numbers (33).

**Figure 8:**
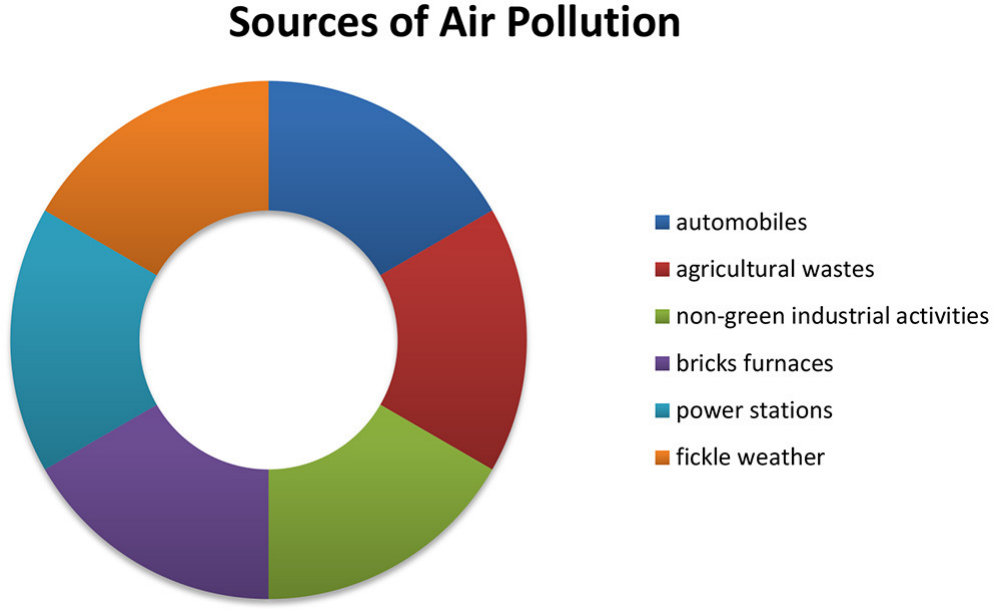
Sources of air pollution in Pakistan.

The reason for air pollution is considered the emanations of automobiles, agricultural wastes, non-green industrial activities, bricks furnaces, power stations, and fickle weather, which pollute the air (Figure 8). It lowered the life expectancy by 2.7 years in Pakistan compared to the WHO 2.2 years depending upon quality air.

This study concludes concerning Pakistan that all industrial, transportation, agricultural, and domestic emissions can lead to respiratory and cardiac risks. The country's policymakers may minimize the respiratory risks by adopting rational decisions and may take help from the Chinese experiment. Since the anti-pollution policy of 2014, air pollution has dropped by 32% (32).

The industries installed at SEZs, automobiles, and other non-green emissions may have the same impact on green growth as those rich GDP countries (26). Then, imagine developing countries and their stagnant economies and the countries of South Asia which are already polluted countries (33). Can they avoid BRI's infrastructure development, trade, and investments negative impact on the environment which may outweigh its economic returns? BRI has direct and indirect impacts on the environment. The greenhouse gas emission from energy projects and transportation of infrastructure may generate more complex risks to the environment [(2), p. 111], which has been concluded in the context of developed countries that emissions may reduce the sustainable development and natural resources (26).

In the BRI package, there are projects related to agriculture and farming, considered the backbone of South Asian economies. However, the new agriculture technologies use much fertilizer, which is regarded as causation of soil degradation. BRI planners for farming must learn from Indian episode where a study showed that most soil erosions, salinity, and general loss of fertile lands occurred due to agricultural fertilizers (27).

Most of the South Asian part of BRI depends on coal power projects which are higher in greenhouse emissions. One respondent mentioned to one of the survey team about its impact on the environment in these words that “I am leading one of these projects, which has a very high emission rate and causing the life of people in danger in surrounding areas. I mean, it is putting peoples' life at stake, but we do not have any proper mechanism in place to handle this. There is a very high emission rate among many other small projects that run under BRI. There are no international standards in place to assess the overall impact of such projects on human life (19).”

#### Impact on Wildlife and Biodiversity

**Figure 9:**
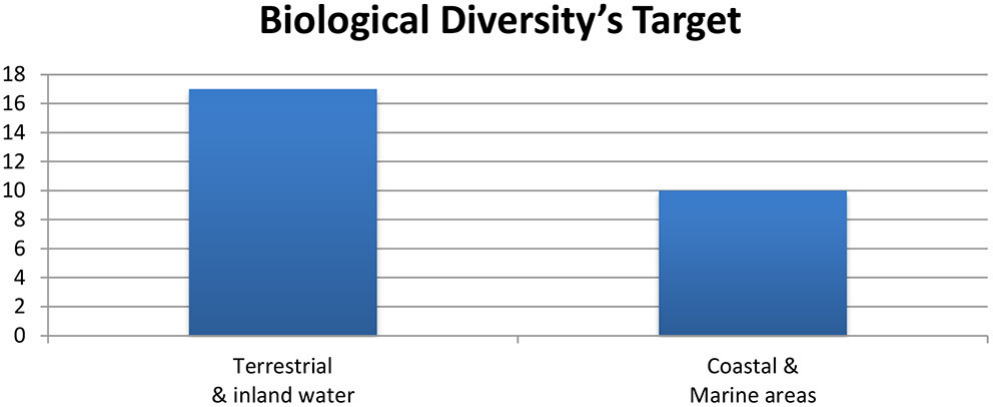
Biological diversity target for the year 2011–2020 (34).

The infrastructure may disturb wildlife's normal movement and matting, which particularly hinders their migration. Construction noise or lightning pollution and deforestation may have an impact on nature. BRI in South Asia needs to consider the timings of matting and migration of wildlife. Retaining trees and hills is necessary for wildlife [(2), p. 118]. BRI in South Asia passes through biodiversity hotspots of Indo-Burma, mountains of Central Asia, Himalaya, Mountains of Southwest China, Indian Ocean, Western Ghats and Sri Lanka, wilderness and conservations areas such as Coral Triangle in Southeast Asia. It is assumed that BRI activities will disrupt biodiversities, such as habitat loss, fragmentations, invasive species, and illegal poaching and logging (34). In the pre-BRI world, fish, birds, mammals, amphibians, and reptiles have decreased 58% since 1970. However, this situation demands urgent action for sustainable future (35). The sea traffic is also contagious to produce water pollution and disruption of invasive species, which consequences may be seen in decades to come, that is why BRI countries must retain the biological diversity convention of protecting 17% terrestrial and 10% coastal and marines (34) (Figure 9).

The traffic not only by land and air will increase, but will also through the sea, because World Trade Organization data for 2018 show that international trade has been increased up to 1,700% in volume by shipping facility and due to the BRI, the further growth in the maritime business is inevitable (36).

Here is another risk of increasing emission of SO_2_; the comparative study of Yangtze River Delta for Shanghai and Suzhou, Ningbo, and Nanjing showed that instead of using the Emission Control Area (ECA)'s Policy, the policy in earlier area is effective up to 1%. In contrast, the latter area did not produce the same results (37). The findings reveal that, in the heterogeneous community of South Asia, the implementation of ECA will be a Herculean job. In the context of BRI, it is observed that the increase in ships traffic will increase ships emission (37).

It may threaten that drinking water, agriculture, and particularly fisheries may face decline due to water pollution (27). To control SO_2_, the International Maritime Organization (IMO) carried out comprehensive legislation, including establishing ECAs and particular navigational areas (37) to reduce ships' emissions. For that, “(38)” introduced more strict rules for minimizing Sulfur in ships' fuel down to 0.50% m/m, which was 3.5% previously. Because IMO concluded that SO_2_ is harmful, may cause respiratory, cardiovascular, and lung diseases to humans, and may also cause acid rain, which may affect crops, forests, aquatic species, and ocean acidification (38).

As it is assumed that the shipping freights will increase under BRI, the empirical data show that out of the world's 49 busiest ports, 16 ports are of China (consult Appendix A). Targeted seaports in South Asia (Port Qasim, Hambantota, Chittagong, and the Maldives) are not fully prepared to handle such heavy traffic from their Chinese counterparts. Otherwise, traffic is likely to increase. The sea freights will bring their benefits and risks, but future emission control is significant for the region (39). Keeping its unprecedented development in mind, WWF concludes that it will substantially impact biodiversity and natural resources (40).

### WWF Policy for BRI

The reports of (35, 40) indicate that BRI may impact biodiversity. That is why the countries need to develop a detailed mapping and analyze it before BRI executions. For this, China is ready to cooperate with partner countries, said by Minister of Foreign Affairs of China (40). WWF reiterated the concept of ecological civilization of China and Sustainable Development Goals (SDGs) signed by 193 UN member countries as policy instruments for BRI partner countries. Governments need to direct significant investment under BRI to ecological and renewable energy infrastructure. WWF is continuously contacting Chinese authorities to make BRI more green [Detailed recommendations (40)]. However, countries individually are also required to follow all international environment-related agreements.

### Geographical Coverage

**Figure 10:**
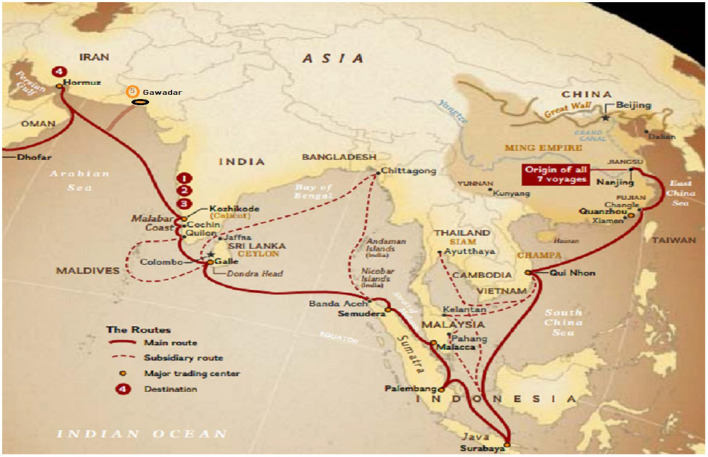
21st Maritime in South Asia [re-developed, Map (68)].

Asia is divided into six regions, that is, (i) the South Pacific, (ii) Northeast Asia, (iii) Central Asia, (iv) West Asia, (v) Southeast Asia, and (vi) and South Asia [(41), pp. 137–138]. This paper is limited to the Geography of South Asia; the countries are Afghanistan, Bangladesh, Bhutan, India, Maldives, Nepal, Pakistan, and Sri Lanka. It covers Silk Road Economic Belt (SREB), and 21st Century Maritime Silk Road. The combination of both is called the BRI. Through Bangladesh-China-India-Myanmar Economic Corridor, Bangladesh, Bhutan, India, Myanmar, Nepal, and Sri Lanka will be connected with China. While through China Pakistan Economic Corridor, Pakistan and Afghanistan will be connected with China (Figure 10).

#### Excessive Use of Natural Resources

Keeping in view one of the aims of BRI is “to build road and rail,” it is understood that it will build an extensive infrastructure, which will take much raw material from the host countries. Besides, other sources such as water and natural sources will be used in huge volumes, which may minimize the sources of energy (19).

#### Production of Massive Waste

One of study in the South Asian context defined waste management in these words “garbage, refuse, sludge from a wastewater or water treatment plant, or air pollution control facility and other discarded material, resulting from industrial, commercial, mining, and agricultural operations, and community activities (42).” Our study added with that definition the classification of waste by Lauritzen, 1994 (a) extraction and processing of raw material, (b) production of building material, (c) construction waste, (d) waste from maintenance and repair, and (e) demolition waste (43).

**Figure 11:**
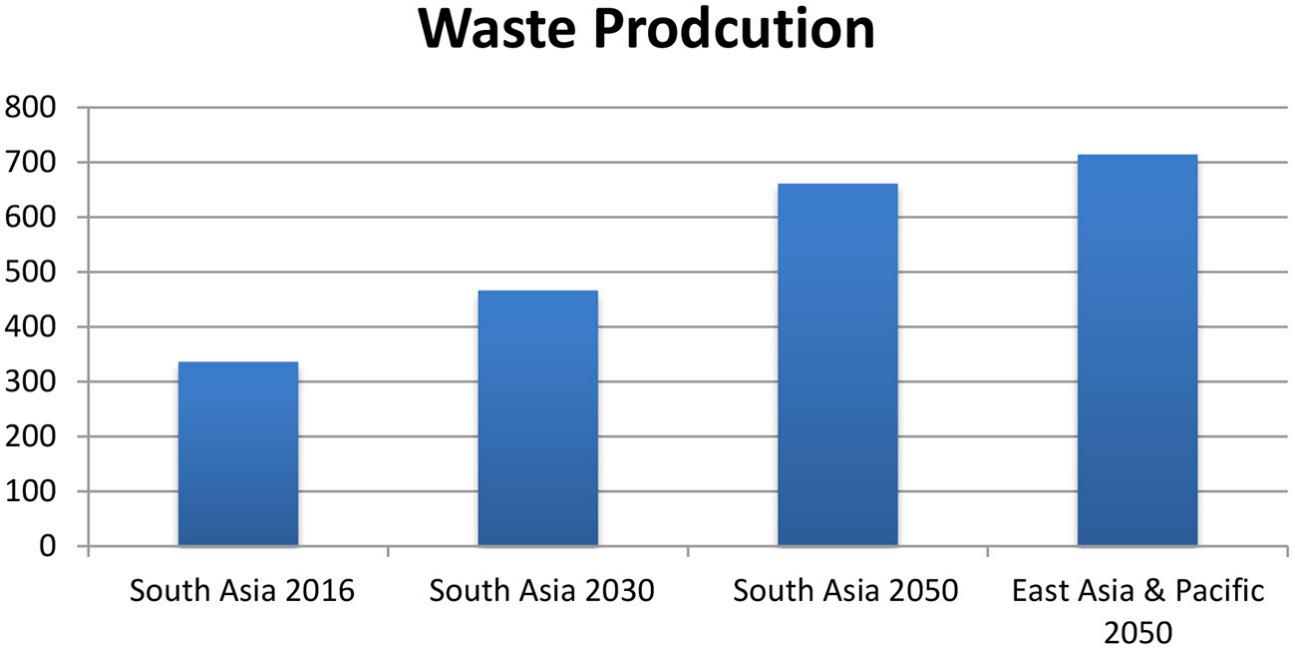
Reproduced with the help of World Bank data (46).

One study concluded that waste would increase in Asia and Africa because of population growth, migrations, rural and urban developments, and raced for economic development. Globalization and shift of production from developed to developing countries may raise waste of industrialization (44). The waste situation in South Asia is already alarming. It is producing 334 million Metric tons of solid waste, ranking it 3rd in the world waste ranking (45). Waste will be doubled in 2050 reaching 661Mt in South Asia (46) (Figure 11). Excluded the dominant waste which is produced from soil excavation, timber, and reinforcing bars when construction projects are implemented (43).

How waste is hazardous to climate, “one kilo of methane (CH_4_) has similar effects of 28 Kilo Carbon dioxide (CO_2_), and the same value is used for methane emission (47).” Moreover, CH_4_ emission from anaerobic decomposition of organic waste at disposal sites is estimated 3% of total emission in 2010 (44).

According to the World Bank 2018 predictions, South Asia will produce 466 million tons of waste in 2030 and 661 million tons of waste in 2050, which will be second after East Asia and pacific with the total of 714 million of tons (46).

Sources showed that the waste mentioned above would increase, producing risks and opportunities for the South Asian economy and environment (See Increase per year in Table 1). The is lump-sum data and cannot determine how much industrial, constructional, and demolition waste is expected categorically. Except from Indian sources, where the government started the classifying waste based on the rules passed in 2016 (48). What can be the possible risks of non-managed waste? Different studies come up with multiple warnings like one study mentioned that it can damage public health, environment, business, tourism, industrial productivity, and economy. The Global Waste Management Outlook (GWMO) mentioned that “the economic loss to society of inaction are 5-10 times higher than the financial costs of proper waste management (44).” Some countries are trying to burn municipal solid waste, another source of hazardous CO_2_, CO, SO_2_, and NO_2_ (49). However, there is a popular view that BRI infrastructure will affect the environment negatively by affecting air from solid waste.

**Table 1:** South Asian Countries waste Production per year.

**Sr. No**	**Country Name**	**Solid waste per year**	**Increase per year**
1	Pakistan	49.6 Mt (2021)	2.4% (62)
2	Bangladesh	78 Mt 2025 (2014)	6.4% (63)
3	India	62 Mt (2018)	4% (64)
4	Nepal	2231 Mt (2020)	6.1% (65)
6	Afghanistan	44 Mt (2016)	(48)
7	Sri Lanka	4.6 billion	3.5% (66)
8	Maldives	365,000 Mt (2017)	0.8% (67)
9	Bhutan	52 Mt 2016	1.5 % (48)

*This table is developed with the help of online sources mentioned in the table*.

## Environmental Impact Assessment and BRI

Environmental impact assessment is a set of principles with some variations from country to country, where countries seek upfront approval of any constructional proposed project, for its ecological and biological effects, on biodiversity, soil, water, air climate, and landscape (50). In Pakistan, EIA was introduced in 1983 and was further strengthened by the Pakistan Environment Protection ACT of 1997, but despite its Regulation of 2000, it did not evolve satisfactorily. The reasons have been highlighted by a study “inadequate capacity of EIA approval authorities, deficiencies in screening and scoping, poor EIA quality, inadequate public participation and weak monitoring in Pakistan (30, 51).” It is anticipated that EIA will provide information about the new projects, programs, policy, or plans through a systematic procedure, which project developers and administrative cadres for quality decisions by identifying its impact on the environment (30). However, it is not available in any research on EIA within Pakistan that it has been considered for CPEC in advance; however, the Ministry of Climate claims that it participated in the meetings of the Shanghai Cooperation Organization (SCO) to ensure environmental resilience for CPEC [(52), p. 14]. Environmental Protection Agency (EPA) has been working since 1997, and to date, February 2022, it has published online more than four-yearly books and 26 environmental impact assessment hearings and reports but none of the activities mentioned CPEC or its related projects giving NOC (53). However, ministry reported its staff meetings in CPEC-related activities (54). However, EIA is ongoing process and EPA of Pakistan may need to mention in their review about the environmental impact of CPEC-related projects, to guide the related ministries on time, and avoid maximum damage of unseen hazardous installations.

Contrary to that, Nepal is taking very detailed EIA and governmental approvals (55) that was observed during BRI project selection when Nepal had a Choice of 36 proposed projects, but out of that after meticulous considerations and committees meetings, Nepal only selected nine projects (56). Comprehensive EIAs and SEAs take time to develop and can result in changes to the original plan, “all of which can lead to project delays. China and BRI partner countries are often reluctant to do anything that could slow projects progress (3).” Interestingly, discussion with many respondents reveals that the BRI will make it extremely difficult for firms to conduct proper environmental assessments. Respondents further contended that China “since the inception of the BRI and even till now has issued very robust and stringent requirements of proper environmental standards. However, many firms have failed to adhere to such stringent requirements imposed in the third-party BRI countries (19).”

Some studies are of the view that BRI excluded the environmental component of the project by excluding EIA, and Environmental Regional Trade Agreements (ERTA). While the earlier employed in many developmental projects across the globe, and it is believed that EIA in the context of Afghanistan is haphazard or unknown (29). However, another study shows that EIA and Strategic Environmental Assessment (SEA) have less consideration (22). As the Chinese Ministry of Ecology and Environment, with the help of ten other departments, including the Environment Protection Bureau carries on the Environmental Impact Assessment. which they divide into three categories of A, B, and C. The project gets A means that it has significant adverse environmental impacts, B represents the plan has limited environmental impacts, and C means a project with no ecological impact (55).

On Chinese land, the phrases such as “Guidance on promoting a Green Belt and Road” and “The Belt and Road Ecological and Environmental Cooperation Plan” one may hear, which promote a very strong pro-environment narrative, along with sub-slogans such as low-carbon development, protect biodiversity, and addressing of climate change (3).

In respect to China Green BRI, it uses all available international and transnational podiums to build networks for environmental protection, such as China-ASEAN, Euro-Asia Economic Forum (EAEF), SCO, Mekong Cooperation, 17+1 cooperation, and 17 Central, and Eastern European countries (3) but what about South Asia, did China used this platform for environmental protection in South Asia. To date, there is no evidence of Pakistan-China Environmental Cooperation Center, Bangladesh-China Environmental Cooperation Center, Sri Lanka- China Environmental Cooperation Center, Afghanistan-China Environmental Cooperation Center, and so on. At the same time, on the other hand, there are Cambodian, Laos, and African-China Environmental Cooperation Centers (3).

## Green Technology and Its Importance

Green Technology, Clean Technology (Cleantech), sustainable technology, also called Environmental Sound Technology (EST), are the combination of products, means, and ways that are environment friendly or, in other words, have minimum or zero contribution to pollution. The usage of GT may control hazardous substances or convert that into harmless substances. By a potential technology to reduce waste, cut pollution, produce green chemicals (green laundry detergent), recycle products, create green energy (from solar), and minimize fossil fuel usage (57). Countries such as Australia, Canada, China, France, Germany, Japan, Norway, South Korea, the UK, and the US are moving toward green hydrogen as an alternative source for energy, which has the lowest emission source (58). One of the studies reworded green economy in these words “Green Economy is a framework of efficient resources of lower carbon emission, less damaging to the environment and more socially inclusive of societies (59).”

Investors around BRI in South Asia should know that green inventions and clean technologies are demanding businesses in days to come, particularly near the BRI route. It will be beneficial for South Asian countries economically and environmentally to have GT because, on the one hand, it will bring investment and technologies to the area. In contrast, it will produce ecologically friendly products and environmental sensitization. BRI countries of South Asia may use solar cells for energy purposes for the route and its related activities, such as lightning and charging. They are providing a reusable water bottle (because GT products demand is on the rise), installing solar water heaters for kitchen and bathing use, and for cleaning water. Installing wind generators at high altitudes at BRI routes and installing rainwater collectors are some of the environmental friendly energy production and natural resources preservation techniques for BRI countries (57). South Asian economies may establish Climate Innovation Center (CIC), and it is an intervention of green business, “builds innovation sites to ameliorate small business start-ups allies to the application of GT, as established at South Africa (59),” which can foster business around BRI in an environmental friendly approach. The mutual relationship between GT and BRI is relatively straightforward. The latter's focus is overall development, while the earlier covers transportation, energy, water, material, productions, and services (60). These sectors are also under the direct focus of BRI in partner countries.

## Findings

It is found that Pakistan will emit more CO_x_, NO_x_, SO_x_, hydrocarbons, and dust particles because of brick kilns coal use. The emission will increase above 34.3% because Pakistan planned to construct more coal-based plants under CPEC, which will harm soil, plants, and amenity. The Ministry of Environment of Pakistan concluded that if the heat increase has not been stopped, the fall of crops and their lifecycle is in danger. Soon, southeastern Pakistan will be unable to produce wheat. However, innovation can minimize industrial process emissions and energy consumption of the construction component of manufacturing by automated solutions, reducing risks to health, environment, and economy. Our study finds that to minimize the heat produced by the construction, one can use digitalization in construction at the workplace and constructional activities. It is the responsibility of both the host and partner countries of BRI in South Asia to adopt biodiversity-friendly policies while implementing the corridor activities or consider the recommendation of WWF for BRI (consult BRI and WWF section).

## Suggestions

The solution to all environmental problems is in adopting a green growth strategy; a study on higher GDP countries concluded that green growth (ecological protection) provides a pathway to combat environmental issues and the use of natural resources (26). Otherwise, climatic changes and environmental degradation will affect Belt and Road Developmental plan in all South Asian countries. If all the participant countries are genuinely after reducing poverty, they must consider the climatic and environmental hazards of the BRI (27). This study suggested that the mentioned countries manage their economic activities in ecological ways. Some authors relate it to an alternative source with the help of technology, “which may reduce fossil fuels and demonstrate less damage to the human, animal, plant health (61).” BRI partner countries must use GT, which is beneficial in reducing waste and pollution from production and consumption. BRI can avoid an environmentally sensitive area by an alternative route [(2), p. 118].

To halt the increase in waste production, sound waste management is required among the South Asian countries, first to focus on regulations and reforms and then partnerships, co-financing, and development of toolkits for global waste management, because local or national government cannot tackle it, it needs an international all over the coordinated plain, which may be supported by international aid or climate funds (44). BRI majority activities may become the reason for creating more heat and environmental issues because of its projects related to energy, transportation, constructions, Industrial activities, agriculture, and food (consult Appendix B). Here, we have taken CPEC as a sample for Bangladesh, China, India, Myanmar (BCIM) Economic Corridor, and Nepal-China-Trans-Himalayan Multi-dimensional Connectivity Network. Bellow pie chart indicates that Pakistan will produce more environmentally hazardous gases by installing 83% of mentioned industries in the coming days. Without bellow mentioned industry, Pakistan is on 142 on EPI list out 180 countries (18).

**Figure 12:**
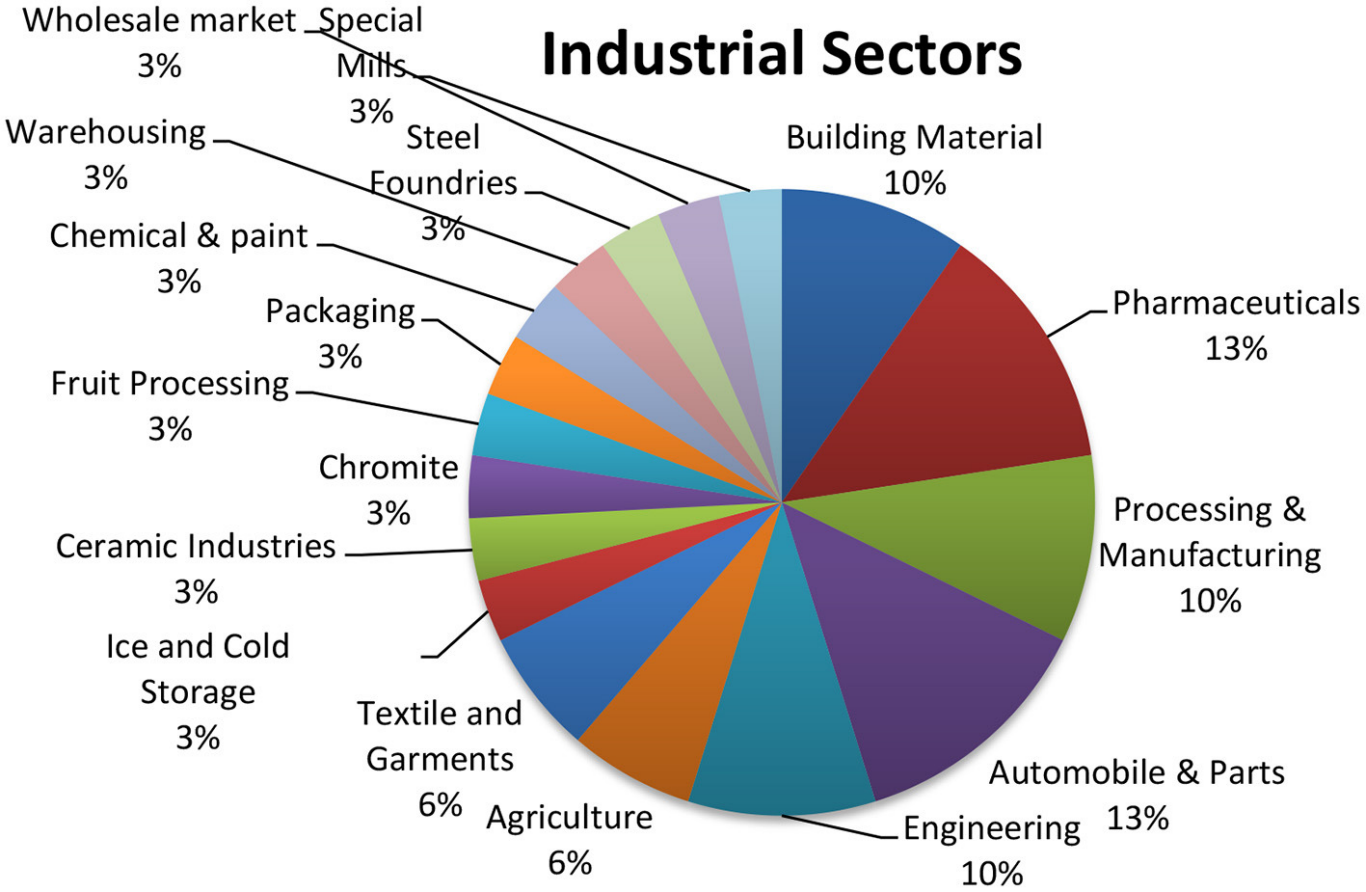
Expected industries SEZs in Pakistan (consult Appendix B for detail).

Figure 12 is developed with the help of the available list of industries on the CPEC official Website, but the data are overlapping and repetitive that is why we segregated data in the sectors as mentioned above and placed it into 14 different categories. There are eight countries on BRI in South Asia; if all of them establish 4 SEZs with the mentioned industry, then the graph of the polluted environment of South Asia will grow stronger than anticipated by World Bank. Here, we excluded Power Generation and Infrastructure, which differ from country to country. It is learned from Central Asian reports and studies that Governments of South Asia should ensure that the roads are appropriately located, designed, and maintained in mountainous areas, and the focus should be on sustaining environmental attributes as compared to economic benefits of local people (29).

If the project is implemented without proper EIA, then it may pollute water and air. Due to deforestation, wildlife and herbs are prone to vulnerability, and it may cause natural calamities.

## Conclusion

**Figure 13:**
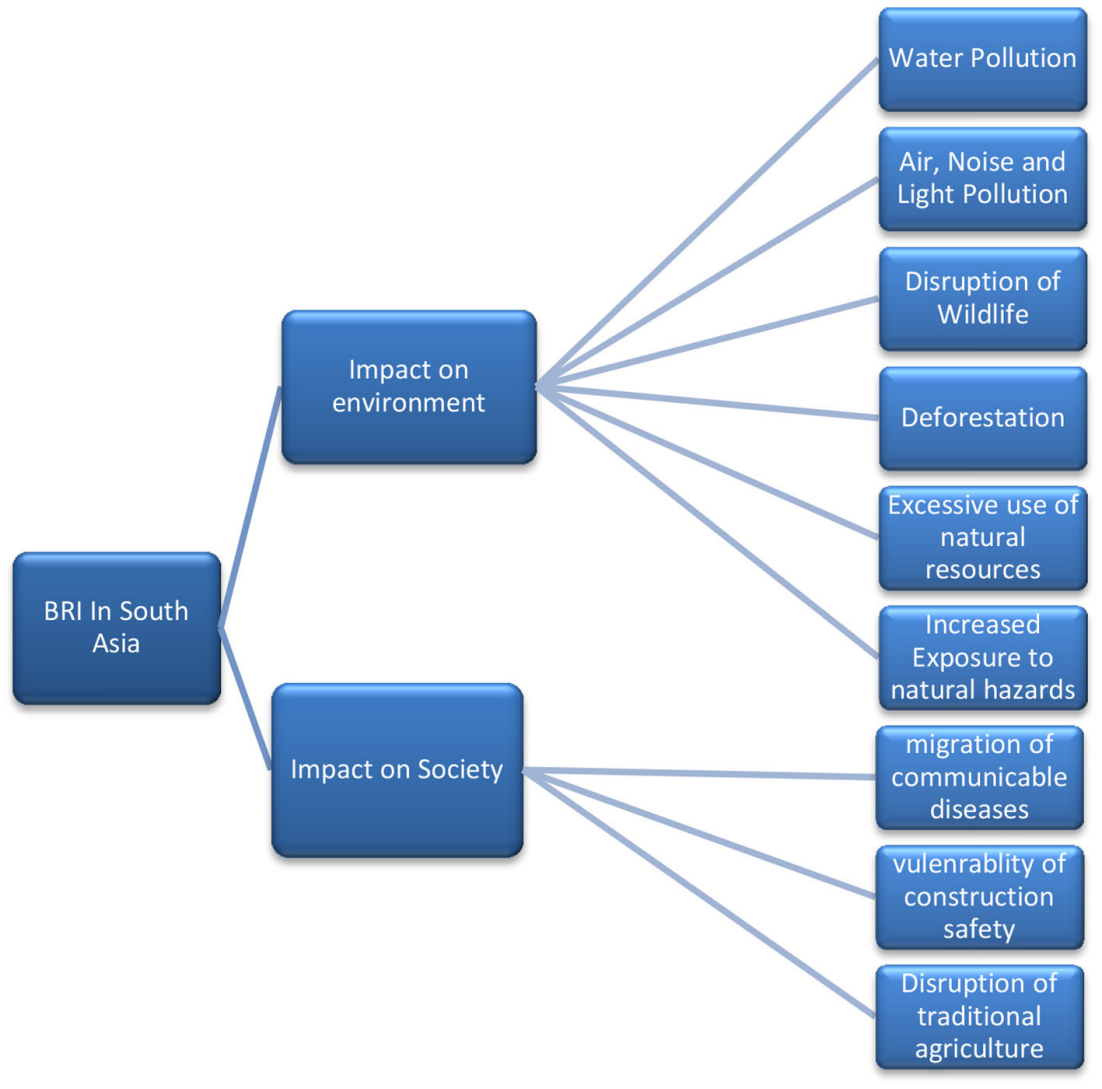
Conceptual representations of How BRI in South Asia can affect the society and environment by heavy constructions and repair of roads, lines, excavations, wastes, building erections, air ports, and sea port constructions.

South Asia is in dire need of infrastructure and construction for economic development, and China is filling that gap through BRI; however, this study finds that it will increase heat, air pollution, noise pollution, water pollution, and deforestation, disruption of traditional agriculture, exposure to natural disasters, and migration of communicable diseases (Figure 13). These mentioned risks can be mitigated by two simultaneous approaches to ensure EIA for every proposed project under BRI and maximize GT's usage. China must peruse South Asian partners to fulfill the requirements for the BRI project under the Paris agreement. Our study gives empirical evidence that in the future, a percentage of emission is expected from the construction around BRI, but we cannot guess the actual amount of percentage because of no availability of factual data about buildings, companies, housings, and other construction within SEZs of South Asia (consult Appendix A, where only names are mentioned, but there is no number). Other initiatives of south Asia such as BCIM and Trans-Nepal and Himalayan corridors may follow the same environmental implications unless adapted to the EIA toolkit. Hypothetically assume that all eight countries of South Asia have full swing operational BRI, then image consumption of energy. Is it increasing or decreasing? If it increases the usage of energy, then, without doubt, it is decreasing green growth.

## Data Availability Statement

The original contributions presented in the study are included in the article/Supplementary Material, further inquiries can be directed to the corresponding author.

## Author Contributions

KF profounded the experience in technicality and placement of ideas and guided MA from time to time. MA developed the conception and design of the study. BH collected the relevant data with the help of NN. NN and BH interpreted some statistical charts. KS helped in drafting and redrafting. All authors contributed to the article and approved the submitted version.

## Conflict of Interest

The authors declare that the research was conducted in the absence of any commercial or financial relationships that could be construed as a potential conflict of interest.

## Publisher's Note

All claims expressed in this article are solely those of the authors and do not necessarily represent those of their affiliated organizations, or those of the publisher, the editors and the reviewers. Any product that may be evaluated in this article, or claim that may be made by its manufacturer, is not guaranteed or endorsed by the publisher.
