# Supplementary material for: Belt and Road Environmental Implications for South Asia
Source: Front Public Health. 2022 Apr 25;10:876606. doi: 10.3389/fpubh.2022.876606 (PMC9084410; doi:10.3389/fpubh.2022.876606)
Supplement: Supplementary file 1 [file Data_Sheet_1.docx]

**Appendix (A) World Top 49 Container Ports**

| **S.N** | **Port** | **Volume 2020 (Million TEU)** | **Volume 2019 (Million TEU)** | **Volume 2018 (Million TEU)** | **Volume 2017 (Million TEU)** | **Volume 2016 (Million TEU)** |
| --- | --- | --- | --- | --- | --- | --- |
| 1 | Shanghai, China | 43.5 | 43.30 | 42.01 | 40.23 | 37.13 |
| 2 | Singapore | 36.6 | 37.20 | 36.6 | 33.67 | 30.9 |
| 3 | Ningbo-Zhoushan, China | 28.72 | 27.49 | 26.35 | 24.61 | 21.6 |
| 4 | Shenzhen, China | 26.55 | 25.77 | 27.74 | 25.21 | 23.97 |
| 5 | Guangzhou Harbor, China | 23.19 | 23.23 | 21.87 | 20.37 | 18.85 |
| 6 | Busan, South Korea | 21.59 | 21.99 | 21.66 | 20.49 | 19.85 |
| 7 | Qingdao, China | 22.00 | 21.01 | 18.26 | 18.3 | 18.01 |
| 8 | Hong Kong, S.A.R, China | 17.95 | 18.30 | 19.6 | 20.76 | 19.81 |
| 9 | Tianjin, China | 18.35 | 17.30 | 16 | 15.07 | 14.49 |
| 10 | Rotterdam, The Netherlands | 14.35 | 14.82 | 14.51 | 13.73 | 12.38 |
| 11 | Jebel Ali, Dubai, United Arab Emirates | 13.5 | 14.11 | 14.95 | 15.37 | 15.73 |
| 12 | Port Klang, Malaysia | 13.24 | 13.58 | 12.32 | 13.73 | 13.2 |
| 13 | Xiamen, China | 11.41 | 11.12 | 10 | 10.38 | 9.61 |
| 14 | Antwerp, Belgium | 12.04 | 11.10 | 11.1 | 10.45 | 10.04 |
| 15 | Kaohsiung, Taiwan, China | 9.62 | 10.42 | 10.45 | 10.27 | 10.46 |
| 16 | Dalian, China | 6.54 | 10.21 | 9.77 | 9.7 | 9.61 |
| 17 | Los Angeles, U.S.A | 9.2 | 9.30 | 9.46 | 9.43 | 8.86 |
| 18 | Hamburg, Germany | 8.7 | 9.30 | 8.73 | 8.86 | 8.91 |
| 19 | Tanjung Pelepas, Malaysia | 9.85 | 9.10 | 8.96 | 8.38 | 8.28 |
| 20 | Laem Chabang, Thailand | 7.55 | 8.10 | 8.07 | 7.78 | 7.22 |
| 21 | Keihin Ports, Japan |  | 8.00 |  | 7.98 | 7.61 |
| 22 | Long Beach, U.S.A. | 8.11 | 7.63 | 8.09 | 7.54 | 6.8 |
| 23 | Tanjung Priok, Jakarta, Indonesia | 6.17 | 7.6, 6.81** | 7.64 | 6.09 | 5.51 |
| 24 | New York-New Jersey, U.S.A. | 7.59 | 7.40 | 7.2 | 6.71 | 6.25 |
| 25 | Colombo, Sri Lanka | 6.85 | 7.23 | 7.05 | 6.21 | 5.73 |
| 26 | Ho Chi Minh City, Vietnam | 7.20 | 7.22 | 6.33 | 6.16 | 5.99 |
| 27 | Suzhou, China |  | 6.27 |  |  |  |
| 28 | Piraeus, Greece | 5.44 | 5.67 | 4.91 | 4.15 | 3.73 |
| 29 | Yingkou, China | 5.67 | 5.48 | 6.5 | 6.28 | 6.08 |
| 30 | Valencia, Spain | 5.43 | 5.44 |  |  |  |
| 31 | Manila, Philippines | 4.43 | 5.31 | 5.05 | 4.82 | 4.52 |
| 32 | Taicang, China |  | 5.15 |  |  |  |
| 33 | Hai Phong, Vietnam |  | 5.13, 4.94 |  |  |  |
| 34 | Algeciras, Spain | 5.11 | 5.12 | 4.77 | 4.39 | 4.76 |
| 35 | Jawarharlal Nehru Port (Nhava Sheva), India | 4.68 | 5.03 | 5.05 | 4.83 | 4.51 |
| 36 | Bremen/Bremerhaven, Germany |  | 4.87 | 5.42 | 5.51 | 5.49 |
| 37 | Tanger Med, Morocco | 5.77 | 4.8 | 3.47 | 3.31 | 2.96 |
| 38 | Lianyungang, China |  | 4.78 | 4.75 | 4.72 | 4.7 |
| 39 | Mundra, India |  | 4.73 | 4.44 | 4.24 | 4.8 |
| 40 | Savannah, U.S.A | 4.68 | 4.59 | 4.35 | 4.05 | 3.64 |
| 41 | Tokyo, Japan |  | 4.51 | 4.57 | 4.5 | 4.25 |
| 42 | Rizhao, China | 4.86 | 4.50 | 4 | 3.24 | 3.01 |
| 43 | Foshan, China |  | 4.44 |  |  |  |
| 44 | Jeddah, Saudi Arabia |  | 4.43 | 4.12 | 4.15 | 3.96 |
| 45 | Colon, Panama | 4.43 | 4.38 | 3.89 | 3.89 | 3.26 |
| 46 | Santos, Brazil |  | 4.17 | 4.12 | 3.85 | 3.6 |
| 47 | Salalah, Oman |  | 4.11 | 3.39 | 3.94 | 3.32 |
| 48 | Dongguan, China |  | 4.00 | 3.5 | 3.91 | 3.64 |
| 49 | Guangxi Beibu, China |  | 3.82 |  |  |  |

Table 2 Data: (World-Shipping-Council, 2019)

**Appendix (B) CPEC Energy related activities under construction**

| Name of Project | Nature of Work | Status by 2022 |
| --- | --- | --- |
| Rashkai Special Economic Zone | 1. Processing and Manufacturing 2. Home Appliances 3. Pharmaceutical 4. Home Building Materials 5. Automobile and parts 6. Agriculture and Horticulture 7. Wholesale Market/ Specialty mills | Complete |
| Dhabeji Special Economic Zone | 1. Light Engineering 2. Automotive and Auto parts 3. Chemical & Pharmaceuticals 4. Consumer Electronics Engineering 5. Textile & Garments 6. Steel-Foundries 7. Warehousing 8. Building Material | Land allocated and bidding in process |
| Allama Iqbal Industrial City | - Textiles - Engineering - Electrical & Electronic - Chemical & Paints - Food Processing - Pharmaceuticals - Automobiles - Packaging - Building Material | Land allocated and bidding and construction in progress |
| Bostan Special Economic Zone | - Fruit Processing - Agriculture machinery - Pharmaceutical - Motor Bikes Assembly - Chromite - Ceramic industries - Ice and Cold storage - Electric Appliance - Halal Food Industry | Land allocated and bidding and construction in progress (Gov-CPEC-PK, 2016) |

References:

1. World-Shipping-Council. The Top 50 Container Ports: These are the Biggest Container Ports in theWorld, the Hubs that Keep Global TradeMoving. (2019). Available online at: https://www.worldshipping.org/top-50-ports
2. Gov-CPECPK. Special Economic Zones. (2016). Available online at: http://cpec.gov.pk/special-economic-zones-projects
